# Genes associated with thermosensitive genic male sterility in rice identified by comparative expression profiling

**DOI:** 10.1186/1471-2164-15-1114

**Published:** 2014-12-16

**Authors:** Yufang Pan, Qiaofeng Li, Zhizheng Wang, Yang Wang, Rui Ma, Lili Zhu, Guangcun He, Rongzhi Chen

**Affiliations:** State Key Laboratory of Hybrid Rice, College of Life Sciences, Wuhan University, Wuhan, 430070 China

**Keywords:** Transcriptome profiling, TGMS, Male sterility transition, Meiosis stage

## Abstract

**Background:**

Thermosensitive genic male sterile (TGMS) lines and photoperiod-sensitive genic male sterile (PGMS) lines have been successfully used in hybridization to improve rice yields. However, the molecular mechanisms underlying male sterility transitions in most PGMS/TGMS rice lines are unclear. In the recently developed TGMS-Co27 line, the male sterility is based on co-suppression of a UDP-glucose pyrophosphorylase gene (*Ugp1*), but further study is needed to fully elucidate the molecular mechanisms involved.

**Results:**

Microarray-based transcriptome profiling of TGMS-Co27 and wild-type Hejiang 19 (H1493) plants grown at high and low temperatures revealed that 15462 probe sets representing 8303 genes were differentially expressed in the two lines, under the two conditions, or both. Environmental factors strongly affected global gene expression. Some genes important for pollen development were strongly repressed in TGMS-Co27 at high temperature. More significantly, series-cluster analysis of differentially expressed genes (DEGs) between TGMS-Co27 plants grown under the two conditions showed that low temperature induced the expression of a gene cluster. This cluster was found to be essential for sterility transition. It includes many meiosis stage-related genes that are probably important for thermosensitive male sterility in TGMS-Co27, *inter alia*: Arg/Ser-rich domain (RS)-containing zinc finger proteins, polypyrimidine tract-binding proteins (PTBs), DEAD/DEAH box RNA helicases, ZOS (C2H2 zinc finger proteins of *Oryza sativa*), at least one polyadenylate-binding protein and some other RNA recognition motif (RRM) domain-containing proteins involved in post-transcriptional processes, eukaryotic initiation factor 5B (eIF5B), ribosomal proteins (L37, L1p/L10e, L27 and L24), aminoacyl-tRNA synthetases (ARSs), eukaryotic elongation factor Tu (eEF-Tu) and a peptide chain release factor protein involved in translation. The differential expression of 12 DEGs that are important for pollen development, low temperature responses or TGMS was validated by quantitative RT-PCR (qRT-PCR).

**Conclusions:**

Temperature strongly affects global gene expression and may be the common regulator of fertility in PGMS/TGMS rice lines. The identified expression changes reflect perturbations in the transcriptomic regulation of pollen development networks in TGMS-Co27. Findings from this and previous studies indicate that sets of genes involved in post-transcriptional and translation processes are involved in thermosensitive male sterility transitions in TGMS-Co27.

**Electronic supplementary material:**

The online version of this article (doi:10.1186/1471-2164-15-1114) contains supplementary material, which is available to authorized users.

## Background

Hybridization is widely used to generate varieties of angiosperms with agriculturally or horticulturally desirable traits. For this, and to optimize hybrid seed production, knowledge of plants’ flowering and other reproductive processes is highly important, and plants with inducible male sterility can be particularly valuable [1, 2]. Analyses of mutants with perturbations of anther development, cloning and functional analyses of the genes involved, and transcriptional studies of key regulatory networks have elucidated many aspects of pollen development [3–8]. A key step is meiosis in the anther, which generates microspores that subsequently develop into mature pollen grains [9].

Hybridization has been particularly valuable for improving yields of the staple crop rice (*Oryza sativa* L.)[10]. For example, hybrid rice lines provide 15–20% higher yields than the best semi-dwarf inbred varieties used in China and elsewhere [11]. Currently, hybrid seeds are usually produced by the cumbersome three-line breeding system. This involves the use of cytoplasmic male sterile (CMS) lines for which limited numbers of appropriate maintenance and restoration lines are available [12]. However, photoperiod-sensitive and thermo-sensitive genic male sterile (PGMS and TGMS, respectively) lines have been recently developed, which allow simpler, cheaper, efficient, safe, two-line hybridization. No maintainer lines are required because PGMS/TGMS lines can serve as both sterile and maintainer lines under appropriate conditions, and negative effects due to sterile cytoplasm or use of cytoplasm from a single line are avoided [13]. Moreover, almost all normal rice strains can restore the fertility of F1 hybrids generated using PGMS/TGMS lines. Consequently, rice breeders can freely choose cross combinations that exhibit strong heterosis to breed super-hybrid rice varieties [14]. Therefore, the two-line system based on PGMS/TGMS lines is increasingly used for hybrid seed production [15].

Numerous researchers have explored the genetic bases of PGMS/TGMS lines and mapped various genes associated with their male sterility on different chromosomes [16–29]. Some of these genes have also been cloned. An example is the *pms3* gene encoding a 1,236-base long noncoding RNA (lncRNA) in the PGMS line NK58S, described as a long-day–specific male-fertility–associated RNA (LDMAR) [30]. Another is *p/tms12-1*, which encodes a small (21-nucleotide) noncoding RNA sequence [31]. It confers PGMS and TGMS in the japonica and indica rice lines NK58S and Peiai 64S (PA64S, derived from NK58S), respectively [31]. A new photoperiod-sensitive genic male sterile line carrying a mutation in the *carbon starved anther* (*CSA*) gene, a R2R3 MYB transcription factor that regulates pollen development, has been subsequently created [32]. Recently, *thermosensitive genic male sterile 5* (*TMS5*) was cloned in AnnongS-1 (AnS-1) [33]. Mutation of this gene causes the TGMS trait through a loss of RNase Z^S1^ (the short-form of RNase Z) function [33]. RNase Z^S1^ can process the mRNAs of *Ub*_*L40*_ (*ubiquitin fusion ribosomal protein L40*), whose over-accumulation causes defective pollen production and male sterility [33]. However, the molecular regulation of the sterility transitions in these lines is not well understood.

We have previously shown that Co27 (renamed TGMS-Co27 here) is a new type of TGMS rice line. A UDP-glucose pyrophosphorylase gene (*Ugp1*) is cosuppressed in the line by over-expression of a *Ugp1*-derived construct in an H1493 background [34]. In the cited study we also showed that UGPase protein accumulated in TGMS-Co27 florets at low temperature, and that the sterility transitions of this line involve temperature-sensitive splicing. However, details of the molecular mechanisms involved are unknown. Therefore, in this work, we compared transcriptomic profiles of meiosis-stage inflorescences in TGMS-Co27 and wild-type (H1493) plants grown at high and low temperatures. The detected differences in expression profiles provide new insights into plants’ responses to temperature changes during reproductive growth and the regulatory networks underlying pollen development in general. In addition, the observed expression profiles of some genes indicated that they are involved in the male sterility transition in TGMS-Co27. Our results represent a source of reference data that should be useful when analyzing the molecular mechanisms underpinning sterility transitions in other PGMS/TGMS rice lines.

## Methods

### Plant materials and growth

Two rice lines were used in this study: the transgenic line TGMS-Co27 and the wild-type japonica variety Hejiang 19 (H1493).

For high temperature cultivation, TGMS-Co27 and H1493 plants were germinated on May 5, grown on May 8 and transplanted on June 3 in an experimental field at Wuhan University Institute of Genetics (Wuhan, China; 30.54°N, 114.36°E). For low temperature cultivation, TGMS-Co27 and H1493 plants were germinated on November 15, grown on November 18 and transplanted on December 12 in a paddy field at Lingshui (18.48°N; 110.02°E), Hainan province. This is a region of southern China that is suitable for growing rice in the winter. All experimental materials were transplanted in the fields with 16.7 cm spacing between plants within rows and 26.7 cm spacing between rows. Meiosis-stage inflorescences of randomly selected plants grown at high and low temperatures were harvested in July and January, when the daily average temperature was 28 and 20°C, respectively, and the photoperiod was 13.5-14 h and 12–12.5 h, respectively.

### RNA isolation and microarray analysis

During the meiosis stage, when florets were 2–3 mm long [35, 36], inflorescences were harvested from both lines under both sets of conditions. Total RNA was isolated using an RNAiso Plus kit (Taraka) following the manufacturer’s recommendations. The transcriptomic profiles of the florets were then explored using standard Affymetrix instruments, protocols and software (obtained from ShanghaiBio Corp.). More specifically, the signal intensities for each probe set on the generated GeneChip microarrays were detected with a GeneChip® Scanner 3000. The raw signals were then analyzed with GeneChip Operating software, with quantile normalization using MAS 5.0 to standardize the distribution of probe intensities for each array in compared sets.

### Microarray data analysis

All transcriptomic data from samples of three biological replicates of both lines grown under both conditions were analyzed by Principal Component Analysis (PCA) using the SBC Analysis System (http://www.ebioservice.com/) and hierarchical clustering (HCL) using MultiExperiment Viewer (Version 4.0). Differentially expressed gene (DEG) analysis was performed using the limma package [37] with fold-change and probability criteria of ≥ 2.0 and < 0.05, respectively. Heatmaps were then constructed using Genesis (Version 1.7.6).

Gene ontology (GO) annotations of detected DEGs were downloaded from the US National Center for Biotechnology Information (http://www.ncbi.nlm.nih.gov), UniProt (http://www.uniprot.org), and GO (http://www.geneontology.org). The elim Fisher algorithm was used to iteratively remove genes mapped to significant GO terms from more general (higher-level) GO terms and and thereby ensure that the former were not overshadowed by the latter [38]. Gene ontology categories with P values < 0.01 were selected.

### Quantitative RT-PCR (qRT-PCR) and semi-quantitative RT-PCR

Total RNA samples (1 μg) from meiosis-stage florets were treated with DNase I (Fementas) and used to synthesize cDNA with oligo(dT) primers and a RevertAid™ First Strand cDNA Synthesis Kit (Fermentas), following the manufacturer’s recommendations. The cDNAs were then amplified by Real-time quantitative PCR (qPCR) using a BioRad CFX96 Real-Time System and SYBR Green PCR Master Mix (Applied Biosystems). Expression levels of all detected sequences were normalized to those of the housekeeping genes *β-actin*, *UBQ5* and *SDHA*. Relative fold expression changes of genes, between lines and conditions, were calculated using the Pfaffl method [39]. Semi-quantitative RT-PCR analysis was performed using a temperature program involving initial denaturation at 94°C for 2 min, followed by an appropriate numbers of cycles (for linear amplification) of 94°C for 30 sec, 55°C for 30 sec and 72°C for 2 min, and then a final extension at 72°C for 10 min. Takara EX Taq™ polymerase was used in all semi-quantitative RT-PCR reactions. The amplified PCR products were resolved by electrophoresis in 1% agarose gels. The RT-PCR products were sequenced to ensure that they were derived from the targeted genes. Primers used in the PCR experiments are listed in Additional file 1.

### RNA gel blot analysis

Total RNA was separated on a denaturing 1.5% formaldehyde agarose gel. Loading of equal amounts of RNA was confirmed by ethidium bromide staining. The RNA was transferred to a Hybond N*+* membrane (Amersham Pharmacia Biotech). *Ugp1* ORF was amplified as probe which was labeled with [α-^32^P]dCTP using the Prime-a-Gene labeling system (Promega), and the membrane was hybridized for at least 10 h at 65°C with the labeled probes. The resulting blots were washed for 15 min at 65°C in 1 *×* SSC and 0.2% SDS followed by 15 min at 65°C in 0.5 × SSC and 0.1% SDS. The membrane was then exposed to storage phosphor screens (Amersham Biosciences), and the hybridization signals were detected using a Typhoon PhosphorImager (Amersham Biosciences).

### Protein extraction and gel blot analysis

Proteins were extracted from meiosis-stage florets, electrophoretically separated and electroblotted. They were then probed immunologically as previously described [34], except that the secondary antibody was goat anti-rabbit IgG (diluted 1:7500 in TBS-T and 1% BSA) conjugated to horseradish peroxidase (HRP). Signals were subsequently quantified by chemiluminescentce using the WBKLS0500 Immobilon Western HRP Substrate (Millipore, USA), BioMax MR X-ray films (Kodak, USA) and a GS-800 scanner (Bio-Rad Laboratories, CA, USA) operated in transmission mode following the manufacturers’ instructions.

### Gene co-expression network analysis

Gene co-expression network analysis was performed to track interactions among the identified DEGs according to their normalized signal intensities in our microarrays. Pearson correlation analysis was applied to each pair of genes and significantly correlated pairs (i.e. Pairs with absolute Pearson correlation coefficients > 0.99) were used to construct the network [40]. In addition, to identify key regulatory genes in the networks, k core-scoring was performed to facilitate subsequent graph topology analysis [41, 42]. The k core-score of a given gene indicates its hub or nodal status with respect to *k* other genes in a network [41, 42]. The genes with the highest k core-scores and degrees of connection were thus identified as “key regulatory genes” in the generated network [43].

## Results and discussion

### Phenotypes and *OsUgp1*expression in TGMS-Co27 and H1493 lines

**Figure 1:**
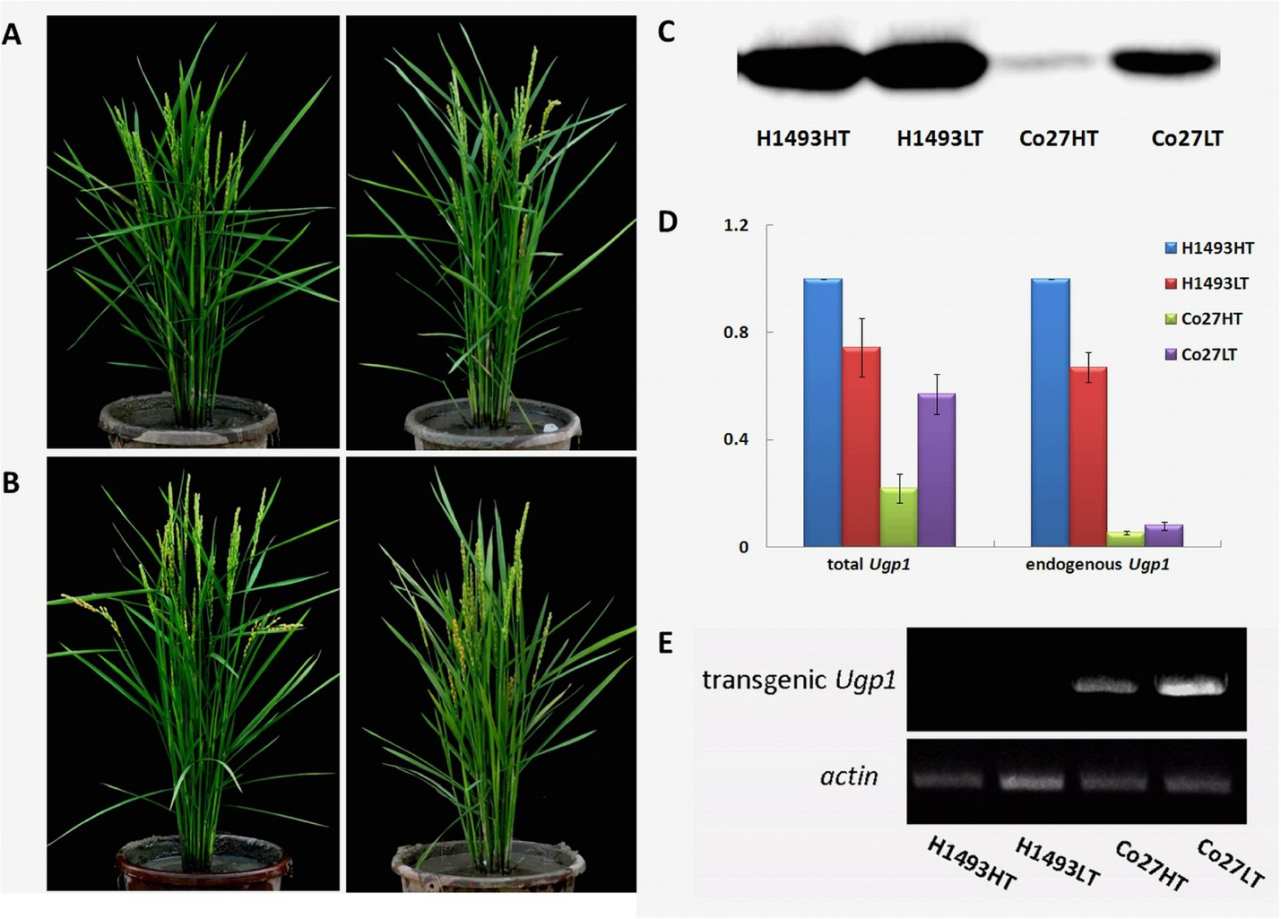
**Phenotypes and** ***OsUgp1*** **expression of H1493 and TGMS-Co27 plants grown at high and low temperature. A**. Phenotypes of TGMS-Co27 plants grown at high temperature (left) and low temperature (right). **B**. Phenotypes of H1493 plants grown at high temperature (left) and low temperature (right). **C**. Results of Western blotting analysis of the abundance of UGPase proteins in florets of H1493 and TGMS-Co27 plants grown at high temperature and low temperature. **D**. Results of qPCR analysis of *OsUgp1* gene expression in the florets. Total *Ugp1* mRNAs include endogenous and transgenic *Ugp1* mRNAs. **E**. Semi-quantitative RT-PCR analysis of the expression of the correctly spliced transgenic *Ugp1* mRNAs. The presence of equal quantities of template in each reaction was verified by amplifying a constitutively expressed actin. H1493HT and H1493LT refer to H1493 plants grown at high temperature and low temperature, respectively. Co27HT and Co27LT refer to TGMS-Co27 plants grown at high temperature and low temperature, respectively.

We previously found that high temperature induces male sterility in TGMS-Co27 by *Ugp1* cosuppression mediated by over-expression of a construct encoding aberrant *Ugp1* transcripts containing the *Ubi1* intron [34]. In the cited study we also found indications that this line’s fertility is restored at low temperature by RNA splicing, which yields *Ugp1* transcripts, albeit at lower than wild-type levels [34]. Accordingly, in the present study TGMS-Co27 was completely male sterile under natural summer conditions in Wuhan but fertile in winter in Lingshui (i.e. the “high temperature” and “low temperature” growth conditions, respectively, as described in the *Materials and methods* section; Figure 1A). In contrast, the wild type line (H1493) was fertile under both conditions (Figure 1B).

Western blotting indicated that fertility reversion at low temperature in TGMS-Co27 is due to accumulation of UGPase protein in florets (Figure 1C). We designed PCR primers targeting the 3’ untranslational region and the open reading frame (ORF) of the *Ugp1* gene to detect endogenous *Ugp1* transcripts and total *Ugp1* transcripts (including endogenous and transgenic *Ugp1* transcripts), respectively. Our qRT-PCR analysis indicated that transcription of the endogenous *Ugp1* was strongly suppressed in TGMS-Co27 florets under both growth conditions. In contrast, expression of total *Ugp1* mRNA was higher at low temperature than at high temperature (Figure 1D). We also designed PCR primers to detect the correctly spliced transgenic *Ugp1* transcripts by amplifying the sequence between the exon of the *Ubi1* promoter and the termination codon of *Ugp1* (Figure 1E). More correctly spliced transgenic *Ugp1* transcripts were accumulated in TGMS-Co27 plants at low temperature than at high temperature. By combining the results of these genetic analyses, we were able to show that the slightly higher expression of total *Ugp1* mRNA in TGMS-Co27 under low temperature conditions is caused by the correctly spliced transgenic *Ugp1* mRNAs. These results are consistent with previous findings [34]. We also unexpectedly detected a slight increase in the expression of total *Ugp1* mRNA in TGMS-Co27 under both conditions. This might be partly due to artefacts arising from partial reverse transcription of longer-than-full-length transcripts and RNA degradation intermediates that were detectable by qRT-PCR but not by semi-quantitative RT-PCR. In accordance with previous studies, the abundance of correctly spliced *Ugp1* mRNAs in TGMS-Co27 florets remained low under both growth conditions and these mRNAs could not be detected by northern blot analysis (Additional file 2: Figure S1) [34]. These findings indicate that both temperature-sensitive splicing and translational regulation may be important for fertility reversion in TGMS-Co27 plants. To identify genes involved in the fertility reversion process, we used Affymetrix microarray analysis to construct transcriptome profiles of florets of both lines grown under both conditions by Affymetrix microarray analysis. Meiosis-stage inflorescences were used for this because pollen mother cells (PMCs) of TGMS-Co27 plants begin to degenerate at this stage [34]. Samples from H1493 plants grown at high temperature, TGMS-Co27 plants grown at high temperature, H1493 plants grown at low temperature and TGMS-Co27 plants grown at low temperature were named H1493HT, Co27HT, H1493LT and Co27LT, respectively.

### Global analysis of the microarray data

**Figure 2:**
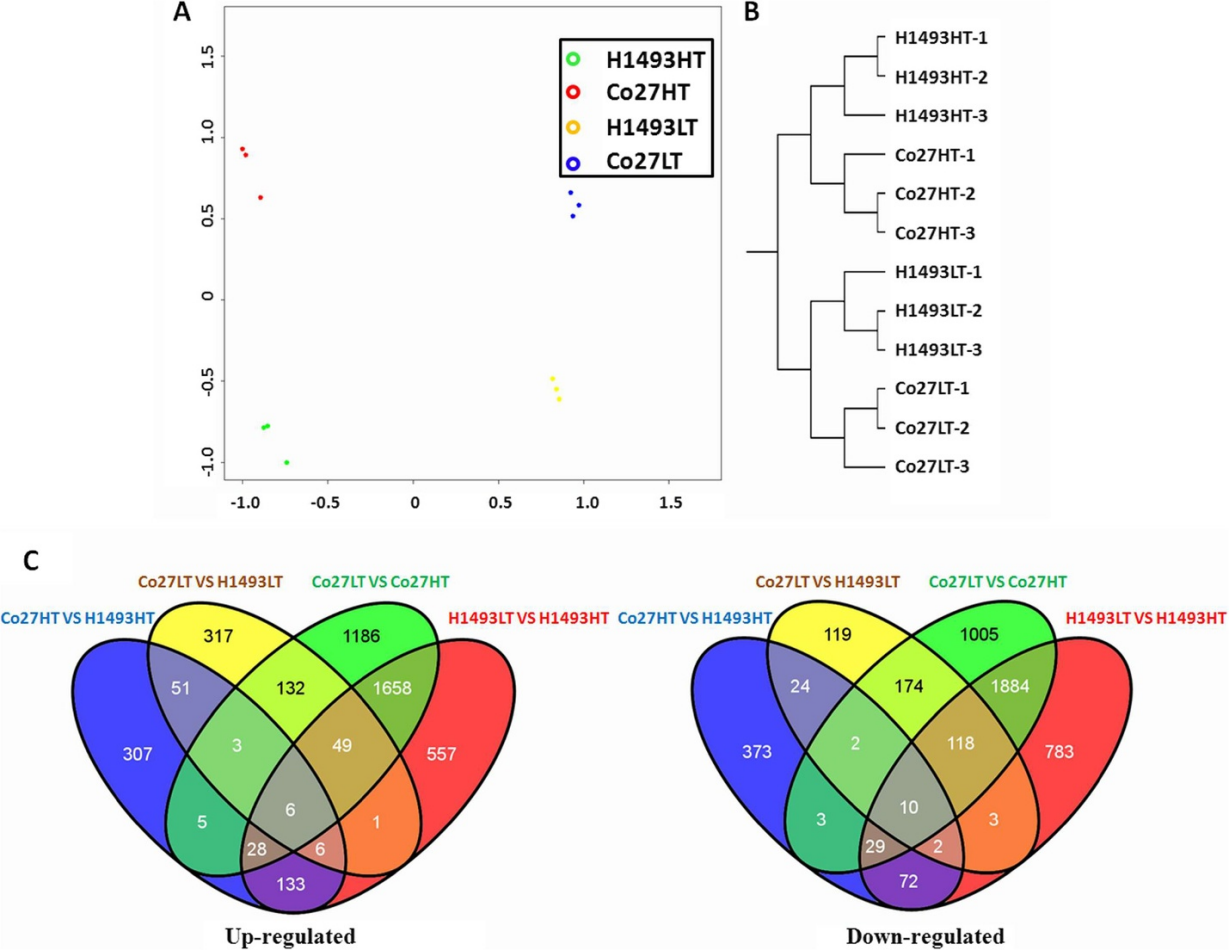
**Global analysis of microarray data.** Figures **A** and **B** show the high reliability and reproducibility of the data. **A**. Principal component analysis (PCA) score plots, and **B**. hierarchical clustering (HCL) of the samples. **C**. Venn diagram of expression profiles of differentially expressed genes (up-regulated and down-regulated) detected in the microarray analysis. The sample names are the same as those in Figure 1C.

Our microarray experiments identified a total of 57,258 probe sets expressed in at least one of the four sets of samples. For quality control, we compared expression files obtained for all of the samples by PCA to ensure that samples representing plants of the same lines grown under the experimental conditions were similar (Figure 2A). Hierarchical clustering (HCL) of the data also indicated clear separation of samples representing different lines and different growth conditions (Figure 2B). Comparisons of the Co27HT vs. H1493HT, Co27LT vs. H1493LT, Co27LT vs. Co27HT and H1493LT vs. H1493HT datasets detected 2581, 2287, 10415 and 8637 probe sets representing 1054, 1017, 6292 and 5339 DEGs with a fold-change (FC) ≥ 2.0 and p-value < 0.05. These observations clearly indicate that the differences between the two growth conditions affected global gene expression more strongly than the genetic differences between the two lines in our four sets of samples. This is consistent with previous findings that temperature affects the expression of far more genes (thousands) than male sterility mechanisms mediated by a single dominant gene (hundreds) [7, 8, 44, 45].

The expression status of the DEGs are illustrated in the Venn diagram shown in Figure 2C. Details of the included DEGs are provided in Additional files 3.

### DEGs between TGMS-Co27 and H1493 at high temperature

**Figure 3:**
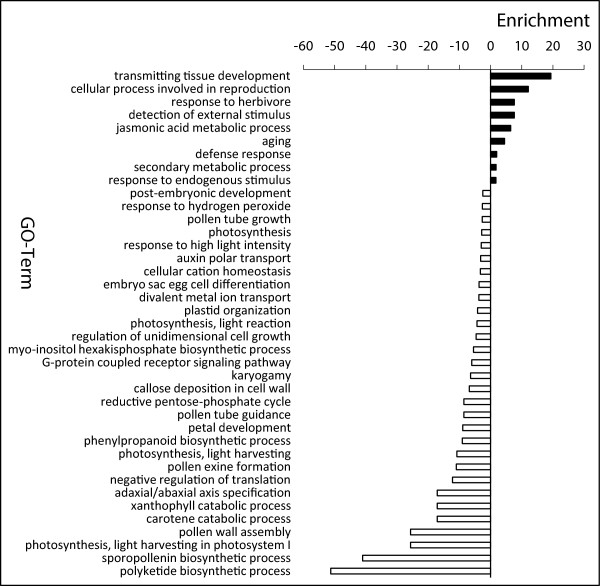
**GO analysis of DEGs between H1493 and TGMS-Co27 at high temperature.** Black and white bars indicate up- and down-regulated genes in Co27HT, respectively.

To elucidate the gene network regulating the temperature-inducible male-sterility of TGMS-Co27, we first examined the 1054 genes that were differentially expressed in TGMS-Co27 and H1493 grown at high temperature (Co27HT vs. H1493HT). There was no significant difference between the numbers of up-and down-regulated genes in Co27HT relative to H1493HT. The functions of the genes exhibiting differential expression between the two lines were investigated by assigning Gene Ontology (GO) categories and enrichments to the up- and down-regulated DEGs (Figure 3; Additional file 4). Based on the assigned GO terms, genes involved in developmental processes (including transmitting tissue development and aging), cellular processes involved in reproduction, responses to stimuli (e.g. responses to endogenous stimuli, detection of external stimuli and responses to herbivores), jasmonic acid (JA)-mediated metabolic processes and secondary metabolic process were enriched in the up-regulated genes. These findings indicate that the absence of normal anther development induces genetic buffering mechanisms via activation of development-, reproduction- and stress-related genes in the male sterile line. The involvement of JA-associated genes is consistent with expectations because in addition to responses to biotic and abiotic stresses, JA also plays pivotal roles in reproduction [46]. Mutants with lesions in various JA biosynthetic enzymes have been shown to be male sterile and defective in pollen maturation and release [47, 48]. In contrast, enrichment of genes involved in many other biological processes (including sporopollenin biosynthesis, pollen wall assembly, pollen exine formation and callose deposition in cell walls) was detected in the repressed sets of genes. These processes are mainly related to pollen development (Additional file 2: Table S1; Additional file 5). UGPase is a key enzyme in carbohydrate metabolism, playing crucial roles in cell wall biosynthesis and callose deposition in cell walls [49]. It also plays a major role in pollen formation and maturation [49], as PMCs and meiotic tetrads are surrounded by callose before meiosis and the formation of the pollen grain wall, respectively [50]. Our microarray, qPCR, northern blotting and western blotting analyses demonstrated that the expression of *Ugp1* (*LOC_Os09g38030*) was much weaker in Co27HT than in H1493HT. Therefore, the expression of these pollen development-related genes may be influenced by low *Ugp1* expression levels. These findings indicate that the anther development regulation network is perturbed in TGMS-Co27 at high temperature.

### DEGs between TGMS-Co27 grown under the two conditions

**Figure 4:**
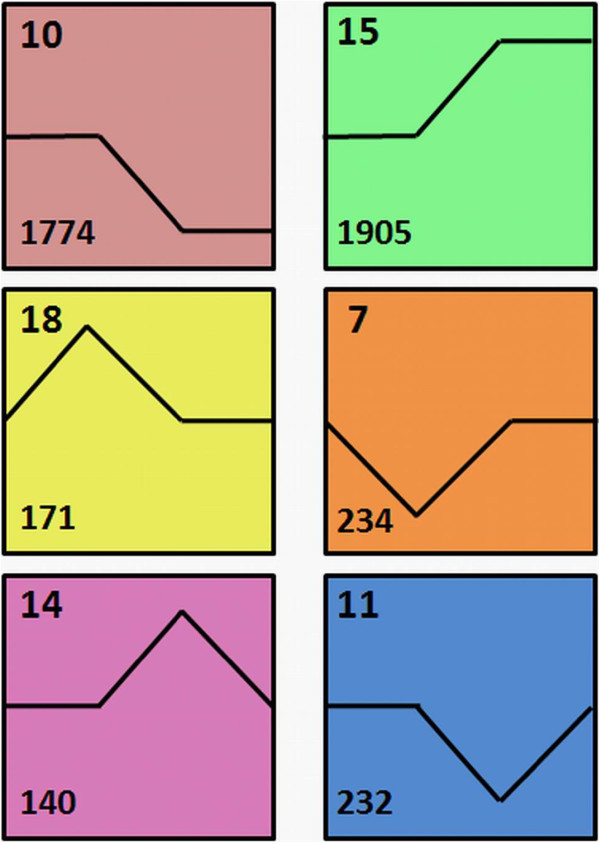
**Significant clusters (p-value < 0.05) of genes differentially expressed between Co27HT and Co27LT.** The four points on the abscissa represent the four sample types, from left to right: H1493HT, Co27HT, Co27LT and H1493LT. The digits in the top and lower left corners indicate cluster numbers and numbers of differentially expressed genes in the corresponding clusters, respectively. The sample names are the same as those in Figure 1C.

To further explore the molecular regulation of sterility transitions in TGMS-Co27, we focused on DEGs between TGMS-Co27 plants grown at high and low temperatures (Co27LT vs. Co27HT). Far more DEGs (10415 probe-sets representing 6292 genes) were detected than in the Co27HT vs. H1493HT comparison, so differences in growth conditions account for most of the significant transcriptomic differences detected. Accordingly, numerous genes were differentially expressed in H1493 under the two growth conditions (8637 probe-sets representing 5339 genes), although there were no obvious phenotypic differences between them. We applied series-cluster analysis to the 10415 probe sets that were differentially expressed in TGMS-Co27 at high and low temperatures to explore the expression dynamics of the corresponding genes in the four sample types. These probe-sets were classified into 25 clusters (Additional file 6), but they were only significantly enriched in seven clusters. Six of these clusters were very significantly separated from the others and could be divided into three pairs of antagonistic clusters (Figure 4). Each of these six contained more than 300 probe-sets.

Clusters 18 and 7 include genes that were either most strongly or most weakly expressed in Co27HT. The cluster 18 sub-set that was highly expressed in Co27HT was enriched in genes associated with stress responses, like the pattern observed in Co27HT relative to H1493HT samples, as discussed in the preceding section (Additional file 7). More excitingly, some genes involved in mRNA catabolic processes were significantly enriched in cluster 7 (Additional file 7). Cluster 11 and 14 were enriched in genes that were most strongly or weakly expressed in Co27LT, and genes related to responses to various stresses and stimuli (e.g. defense, water deprivation and cold responses) (Additional file 7). These findings indicate that expression of some stress-related genes in TGMS-Co27 was perturbed at low temperature as well as high temperature.

Clusters 10 and 15 included the largest number of DEGs (1774 and 1905 genes with GO annotations, respectively), corroborating the broad impact of growth conditions on gene expression. GO assignations of cluster 10 indicated that it was enriched in many genes involved in low temperature response (e.g. lipid transport and responses to cold and oxidative stress) (Additional file 2: Figure S2; Additional file 7). A considerable number of genes related to these stress responses were also found in cluster 15 (Additional file 2: Figure S2; Additional file 7). However, small numbers of photoperiod response-related genes were found in both clusters (Additional file 2: Figure S2; Additional file 7). This is consistent with a previous report that day length only regulated the expression of 100–200 genes in NK58S [51]. Similarly, another study showed that a shift from high to low temperatures caused more pronounced changes in the protein expression patterns of peach trees than did a shift from long to short photoperiods [52]. These results indicated that temperature rather than day length was the main environmental factor affecting global gene expression in our sampled plants. As temperatures at both sites were suitable for rice growth, the detected differences in gene expression indicate that plants may fine-tune their expression profiles via adaptive responses to temperature changes that optimize their growth and reproduction processes. In accordance with this hypothesis, two rice *CBF/DREB* (C-repeat-binding factors/dehydration-responsive element-binding factors) genes, *LOC_Os09g35030* and *LOC_Os06g03670* (homologs of Arabidopsis *CBF2* and *CBF3* genes, respectively), were induced by low temperature. CBF/DREBs are well known cold-responsive transcriptional activators, and their overexpression of CBF/DREBF reportedly increases freezing tolerance in Arabidopsis and rice [53, 54]. These results indicate that the molecular mechanisms involved in plants’ chilling and cooling responses have common elements.

More significantly in the context of this study, cluster 15 was enriched in genes related to DNA replication, repair and recombination; chromosome organization; and cell cycling (meiosis and mitosis), division and proliferation (Additional file 7). This is consistent with the importance of meiosis in the sexual reproduction of rice for formation of haploid spores and gametes. It is also the most sensitive stage to various stimuli because most male fertility perturbations occur during meiosis [55], including perturbations that occur in PMCs in TGMS rice lines. For example, PA64S plants are reportedly completely male sterile when grown at high temperatures (25–30°C), but male fertile when exposed to lower temperatures (21–23°C) during the microspore mother cell (MMC) to meiosis stages [31]. The fertility of many other TGMS rice lines is also temperature-sensitive between the stages of the pistil/stamen primordium-formation and meiosis [56]. Furthermore, combinations of low temperature and long photoperiods or high temperature and short photoperiods may reduce male fertility in PGMS rice plants. Notably, the male fertility of Nongken58S and W6154S (PGMS lines whose male fertility is mainly photoperiod- and temperature-dependent, respectively) is thermosensitive from panicle primordium differentiation and from stamen/pistil primordium formation to the single nuclear pollen stage, respectively [57]. Thus, although reported thermosensitive phases differ among PGMS/TGMS rice lines, they all include the most sensitive meiosis stage and temperature is a common regulator. The low temperature-induced genes in cluster 15 may help PMCs progress to meiosi, so this cluster appears to contain the genes with the greatest impact on PGMS/TGMS processe. This finding will hopefully support detailed investigations into the molecular mechanisms involved in sterility transitions in various other PGMS/TGMS rice lines.

### TGMS related-genes in TGMS-Co27

As already mentioned, TGMS-Co27 plants reverted to fertility and accumulated UGPase protein in their inflorescences at low temperature. While *Ugp1* remained cosuppressed in these plants, the levels of correctly spliced *Ugp1* mRNAs that accumulated in them were far greater than those observed in Co27HT plants, although lower than the wild-type levels. Therefore, we hypothesized that the DEGs between Co27LT and Co27HT involved in post-transcriptional and translational process may have important functions in TGMS. In accordance with this hypothesis, temperature- and/or photoperiod-sensitive post-transcriptional regulation of key transcripts has been detected in other PGMS/TGMS rice lines. For example, processing of LDMAR (see *Introduction*) in Nongken58S and a non-coding RNA expressed from the corresponding locus in the indica line PA64S is involved in their PGMS/TGMS transitions [30, 31]. In addition, RNase Z^S1^-mediated *Ub*_*L40*_ mRNA regulation causes a TGMS trait in *tms5* rice mutants [33]. Temperature- and photoperiod-sensitive translation is also important in various other organisms. For example, temperature-sensitive translational regulation of FREQUENCY (FRQ) contributes to temperature sensing by the circadian clock of *Neurospora crassa* [58, 59], and light-sensitive translational regulation of DOUBLETIME contributes to circadian period modulation in *Drosophila* [60].

As also mentioned, 10415 probe-sets representing 6292 genes were differentially expressed between Co27LT and Co27HT. According to the GO terms in Table 1, 178 probe-sets representing 156 of these DEGs may be directly involved in TGMS-related post-transcriptional and translational processes (Table 1; Additional file 8). In addition, hierarchical clustering (HCL) of the expression profiles of these 178 probe-sets in the four sample types revealed that about two-thirds of them were up-regulated in Co27LT relative to Co27HT (Table 1; Additional file 2: Figure S3; Additional file 8). However, most of these genes were not differentially expressed between TGMS-Co27 and H1493 at either high or low temperature (Additional file 2: Figure S3). We also matched these 156 genes to the 25 clusters we previously identified (Additional file 8). Eighty-six genes were significantly (p = 0.012) included in cluster 15, confirming that this is the most important cluster for fertility alteration in TGMS rice lines. In contrast, 16 genes were included in clusters 7 and 8, which contained genes that were particularly weakly expressed in Co27HT.

**Table 1:** **GO annotations of TGMS-related genes**

GO ID	GO term	Gene number.	
		Up	Down
**GO:0006397**	mRNA processing	15	7
**GO:0000398**	mRNA splicing, via spliceosome	13	10
**GO:0008380**	RNA splicing	11	9
**GO:0031123**	RNA 3'-end processing	2	1
**GO:0000966**	RNA 5'-end processing	1	0
**GO:0006378**	mRNA polyadenylation	1	0
**GO:0043484**	Regulation of RNA splicing	1	0
**GO:0006412**	Translation	58	38
**GO:0006417**	Regulation of translation	12	5
**GO:0006413**	Translational initiation	6	4
**GO:0006414**	Translational elongation	3	3
**GO:0006415**	Translational termination	2	1
**GO:0006446**	Regulation of translational initiation	1	0
**GO:0017148**	Negative regulation of translation	1	1
**GO:0045727**	Positive regulation of translation	3	0
**GO:0045900**	Negative regulation of translational elongation	1	0
**GO:0045901**	Positive regulation of translational elongation	0	1
**GO:0045905**	Positive regulation of translational termination	0	1
**GO:0045947**	Negative regulation of translational initiation	0	1

The up and down gene numbers indicate the numbers of genes that were expressed more strongly in TGMS-Co27 plants at low temperature than at high temperature and vice versa, respectively.

**Figure 5:**
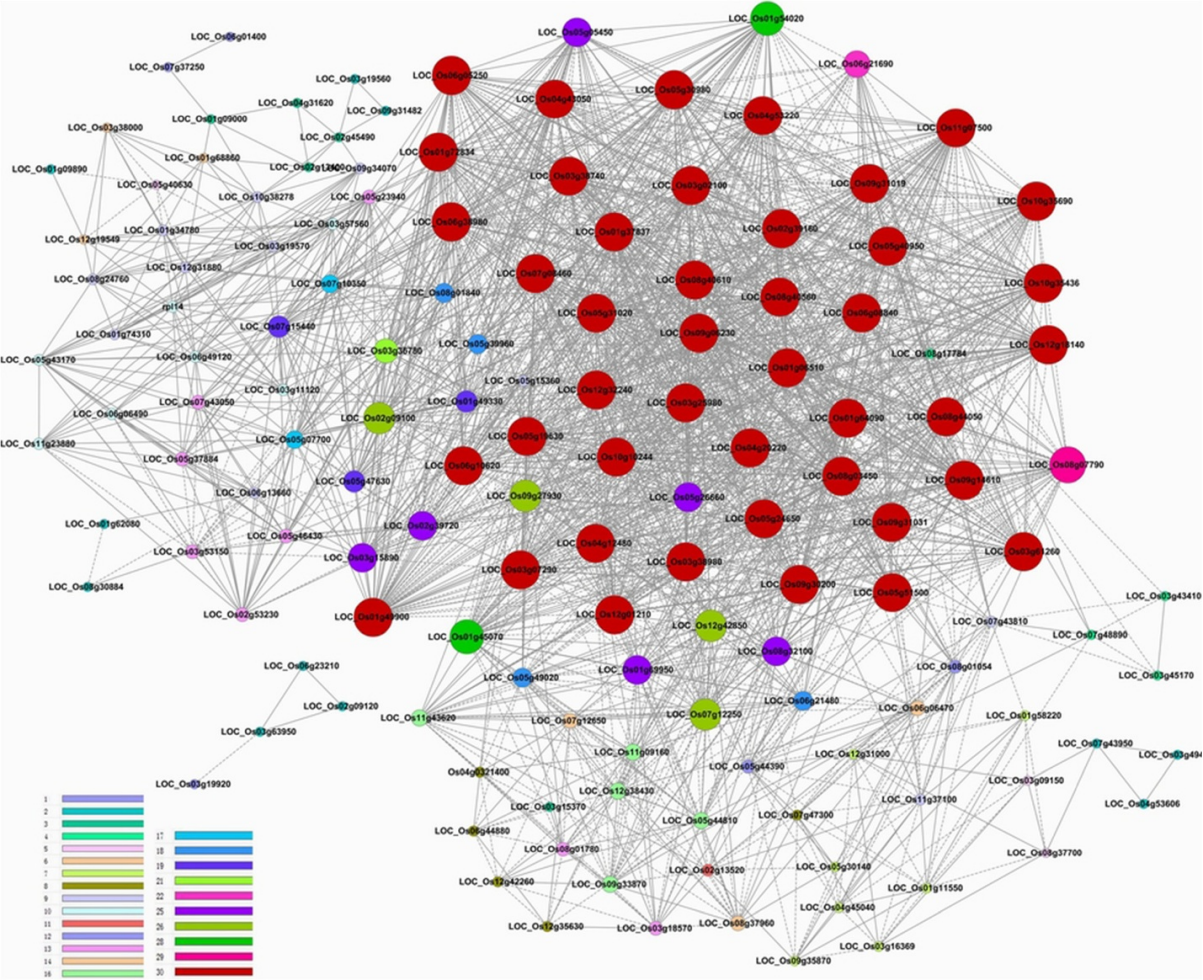
**Co-expression network of the 156 TGMS-related differentially expressed genes.** The color of each circle indicates the k core-scores of the corresponding genes, according to the key in the lower left corner, and its size indicates the k-core score of the genes.

We constructed a gene co-expression network based on the normalized signal intensities of the 156 DEGs in our microarrays (Figure 5; Additional file 9) to examine possible functional linkages among them [61]. The genes’ “k core-scores” (see *Materials and methods*) were used to identify “key regulatory” genes within this set, i.e. genes that have high networking degrees and appear to govern the interactions among the DEGs. Fifty-eight genes were assigned k core-scores exceeding 20 (Additional file 9), 39 and 19 of which were included in clusters 15 and 10, respectively. According to their GO annotations, 11 and 29 genes included in cluster 15 have functions in post-transcriptional and translational processes, respectively, and one participates in both of these processes (Additional file 9).

Post-transcriptional processes are very important in the regulation of eukaryotic gene expression and are associated with numerous plant responses to abiotic stresses [62]. One of the key processes is alternative splicing, which can increase protein diversity and affect mRNA stability [63, 64]. This occurs in the spliceosome, which contains abundant small nuclear RNAs (snRNAs) and about 170 proteins, including various RNA-binding proteins, RNA-dependent ATPases, RNA helicases and protein kinases involved in splicing regulation [64–67].

Two of the 17 “key regulatory” genes (*LOC_Os06g08840* and *LOC_Os02g39720*), which encode Arg/Ser-rich domain (RS)-containing zinc finger proteins (OsRSZp21a and OsRSZp23, respectively), were down-regulated in Co27LT compared with Co27HT. Expression of *RSZpZ23* is also reportedly decreased in *japonica* and *indica* rice, while *RSZpZ21a* is up-regulated in *japonica* rice but down-regulated in *indica* rice, under cold stress [68]. Our results show that *RSZpZ23* was down-regulated in both rice lines at low temperature, but *RSZpZ21a* was down-regulated in Co27LT. RSZp21a, RSZp21b and RSZp23 are three human 9G8-type proteins that have a RNA recognition motif (RRM) and a RS domain separated by a CCHC-type zinc knuckle [68]. RSZp23 has been shown not only to increase splicing efficiency but also to alter the selection of 5’ splice sites of the *Wx*^*b*^ intron and might affect other steps in RNA processing and translation [69]. Serine/arginine-rich (SR) proteins play important roles in both the constitutive and alternative splicing of pre-mRNA [70]. We detected weak expression of these proteins (which may be crucial for the correct splicing of exogenous *Ugp1* transcripts at low temperature) in Co27LT.

In addition, a polypyrimidine tract-binding protein (PTB)-encoding gene (*LOC_Os03g25980*) was up-regulated in both rice lines at low temperature. PTBs are key splicing factors that influence splice site selection and orchestrate coordinated splicing programs during developmental processes. Notably, two Arabidopsis *PTB*-related genes (*AtPTB1* and *AtPTB2*) are reportedly involved in pollen germination [71, 72]. Therefore, stronger expression of the *PTB* gene is likely important for the correct splicing of exogenous *Ugp1* mRNAs and restoring fertility in TGMS-Co27 plants at low temperature. As these genes involved in alternate splicing were temperature-regulated, transcripts of selected SR genes that are known to be alternatively spliced [69, 73] were analyzed to explore the influence of temperature on their splicing patterns (Additional file 2: Figure S4). We found that low temperature had dramatic effects on the splicing pattern of *SR33*, *SCL26* (*SC35-like family*), *SCL30* and *RSZ37*. Although *SCL30a* did not show temperature-related differences in splicing patterns, its expression pattern was very similar to that of *Ugp1*, corroborating the conclusion that *SR* genes may be involved in temperature-sensitive alternative splicing of *Ugp1*. These results clearly indicate that low temperature affects the global splicing of mRNAs.

Two DEAD/DEAH box RNA helicase-encoding genes, *HEN2* (*HUA enhancer 2*; *LOC_Os11g07500*) and *SKIV2L2* (*superkiller viralicidic activity 2-like 2*; *LOC_Os12g18140*), were also up-regulated in Co27LT. *AtHEN2* is highly expressed in *Arabidopsis* inflorescences, specifically in their meristems and developing flowers, where it is apparently required for maintenance of expression of MADS-box genes, class B and C [74]. A homologue of the *SKIV2L* genes in Arabidopsis is *MTR4*, which encodes a HEN2-homologous protein required for proper rRNA biogenesis [75]. Plants lacking AtMTR4 are viable but have several developmental defects, including aberrant vein patterning and pointed first leaves [75]. DExD/H box RNA helicases have multiple functions in transcriptional processes that involve multi-step association/dissociation of large ribonucleoprotein (RNP) complexes and modulation of complex RNA structures, including pre-mRNA processing, ribosome biogenesis, RNA turnover, RNA export and translation [76]. Thus, the higher expression levels of these two genes in Co27LT indicate that they may also be important in the TGMS process.

C2H2 zinc finger proteins (ZFPs) constitute one of the largest nucleic acid-binding protein families in eukaryote genomes. They have diverse functions, from DNA or RNA binding to involvement in protein-protein interactions. ZFPs of *Oryza sativa* are called ZOS and the corresponding genes are numbered in the order of their physical positions on the chromosomes [77]. The TIGR database includes 179 *ZOS* genes with corresponding locus IDs, some of which are regulated by abiotic (e.g. cold, dehydration and salt) stresses [77]. Expression of C2H2 family genes is particularly strong during panicle development in rice [78], and one (*STAMENLESS 1*, *SL1*) is involved in floral development [79]. We found that expression of *ZOS8-11* (*LOC_Os08g40560*) was down-regulated in Co27HT compared with H1493HT, but restored to normal levels in TGMS-Co27 plants at low temperature. Thus, it may have a different mode of action to other genes involved in splicing. Another “key regulatory” gene involved in post-transcriptional processes (*LOC_Os06g38980*), which was up-regulated in Co27LT, encodes a polyadenylate-binding protein. This is an essential, well-conserved, multifunctional protein that is involved in polyadenylation, export of mRNAs to the cytoplasm, translation initiation, translation termination, mRNA biogenesis and degradation [80, 81]. In summary, our results indicate that RS-containing zinc finger proteins, PTBs, DEAD/DEAH box RNA helicases, ZOS, one or more polyadenylate-binding proteins, and some other RRM-containing proteins are important for post-transcriptional processes involved in TGMS (Table 2).

**Table 2:** **Candidate TGMS-related genes**

Query symbol	probeID	Description	E-Value	Subject symbol	logFC_Co27H/HH	logFC_Co27L/ Co27H	logFC_HL/HH
**Splicing**							
**LOC_Os06g08840**	OS.7772.2.A1_AT	OsRSZp21a	5.00E-42	AT4G31580.2	0.14	**-1.02**	-0.8
**LOC_Os02g39720**	OsAffx.2920.1.S1_s_at	OsRSZp23	6.00E-39	AT4G31580.2	0.37	**-1.62**	**-1.4**
**LOC_Os03g25980**	OS.7952.1.S1_AT	Polypyrimidine tract-binding protein	5.00E-172	AT3G01150.2	-0.12	**1.1**	**1.04**
**LOC_Os11g07500**	OS.14143.1.S1_AT	HUA enhancer 2	0	AT2G06990.1	-0.03	**1.4**	**1.4**
**LOC_Os12g18140**	OS.47607.1.S1_AT	Superkiller viralicidic activity 2-like 2	0	AT1G59760.1	-0.16	**1.24**	0.99
**LOC_Os08g40560**	OS.15831.2.S1_AT	ZOS8-11	0	AT2G27100.1	**-2.28**	**2.4**	0.06
	OSAFFX.6095.1.S1_S_AT				-0.87	**1.64**	0.73
	OS.15831.3.S1_AT				0.29	**1.89**	**2.15**
	OS.15831.3.S1_X_AT				0.14	**1.81**	**1.73**
**LOC_Os01g72834**	OS.6252.1.S1_AT	RNA recognition motif containing protein	8.00E-52	AT1G33470.2	-0.18	**1.04**	0.87
**LOC_Os03g15890**	OS.12705.1.S1_AT	RNA recognition motif containing protein	3.00E-37	AT1G07350.2	-0.35	**1.5**	**1.29**
	OS.12705.2.S1_X_AT				-0.17	**1.09**	**1.11**
**LOC_Os05g30980**	OS.8371.1.S1_X_AT	RNA recognition motif containing protein	3.00E-126	AT4G03110.2	-0.19	**1.06**	0.78
	OS.8371.2.S1_AT				-0.16	**1.1**	0.91
**mRNA processing**							
**LOC_Os06g38980**	OS.51905.1.S1_AT	Polyadenylate-binding protein	5.00E-154	AT1G22760.1	-0.5	**1.4**	0.7
	OSAFFX.5057.1.S1_S_AT				-0.04	**1.3**	**1.53**
**Translation initiation**							
**LOC_Os05g51500**	OS.12337.1.S1_AT	eIF5B	0	AT1G76810.1	-0.25	**1.59**	**1.09**
	OS.12337.2.S1_X_AT				0.2	**1.27**	**1.32**
**Ribosomal proteins**							
**LOC_Os08g03450**	OS.50740.1.S1_AT	Ribosomal protein L37	8.00E-41	AT1G15250.1	-0.6	**2.34**	**1.86**
	OS.50740.1.S1_X_AT				0.51	**2.74**	**2.94**
**LOC_Os01g64090**	OSAFFX.2398.1.S1_AT	Ribosomal protein L1p/L10e	0	AT1G08360.1	-0.01	**1.27**	**1.33**
**LOC_Os01g69950**	OS.4997.1.S1_S_AT	Ribosomal protein L27	2.00E-58	AT5G40950.1	0.2	**1.08**	**1.38**
	OS.51456.1.S1_AT				0.02	**1.2**	**1.14**
**LOC_Os07g12250**	OS.14565.1.S1_AT	Ribosomal protein L24	0	AT2G36620.1	0.06	**1.09**	**1.22**
**Aminoacyl-tRNA synthetases**							
**LOC_Os01g06510**	OS.46028.1.S1_AT	Arginyl-tRNA synthetase	0	AT4G26300.1	-0.14	**1.87**	**1.55**
**LOC_Os01g37837**	OS.5952.2.S1_S_AT	seryl-tRNA synthetase, putative, expressed	2.00E-79	AT5G27470.1	-0.1	**1.1**	**1.08**
**LOC_Os01g54020**	OS.14456.1.S1_AT	tRNA synthetase	2.00E-147	AT2G25840.3	-0.41	**1.83**	**1.37**
**LOC_Os02g09100**	OSAFFX.2548.1.S1_AT	Isoleucyl-tRNA synthetase	0	AT4G10320.1	-0.63	**2.66**	2.09
**LOC_Os03g02100**	OS.33311.1.S1_AT	valyl-tRNA synthetase	0	AT1G14610.1	-0.26	**2.01**	**1.71**
**LOC_Os03g38980**	OS.27850.1.S1_AT	tRNA synthetases class II domain containing protein	0	AT3G11710.1	0	**1.19**	**1.2**
**LOC_Os10g10244**	OS.11722.1.S1_A_AT	alanyl-tRNA synthetase	0	AT1G50200.2	-0.13	**1.15**	1
**Translation termination**							
**LOC_Os04g20220**	OS.16925.1.S2_AT	Elongation factor Tu	0	AT1G18070.3	-0.07	**1.01**	0.97
	OS.7254.1.S1_AT				0.01	**1.23**	**1.34**
**LOC_Os05g19630**	OS.7254.2.S1_X_AT	Peptide chain release factor protein	4.00E-141	AT2G47020.2	-0.13	**1.24**	**1.09**
**Other translation factors**							
**LOC_Os09g06230**	OS.46327.1.S1_AT	serine/threonine-protein kinase 16	8.00E-138	AT5G08160.2	0	**1.42**	**1.23**
**LOC_Os05g05450**	OS.18996.1.S1_AT	MA3 domain containing protein	2.00E-159	AT5G17930.1	-0.3	**1.3**	**1.13**
**LOC_Os06g10620**	OS.6853.1.S1_AT	Transcription elongation factor SPT5 homolog 1	0	AT4G08350.1	-0.18	**1.07**	0.97

HH and HL refer to H1493 plants grown at high temperature and low temperature, respectively, while Co27H and Co27L refer to TGMS-Co27 plants grown at high temperature and low temperature, respectively. Significant changes (p-value < 0.05; fold change ≥ 2) are indicated by boldface.

As mentioned, TGMS-Co27 plants accumulated UGPase protein in their inflorescences at low temperature although the *Ugp1* gene was cosuppressed in them and small amounts of correctly spliced *Ugp1* mRNAs were detected by PCR (but not northern blotting). Thus, we predicted that some DEGs between Co27HT and Co27LT involved in translational processes may be important for the accumulation of UGPase protein in Co27LT. In this section, we mainly discuss the “key regulatory” genes of this type that we identified. Translational control of existing mRNAs allows for more rapid changes in cellular concentrations of the encoded proteins than transcriptional or post-transcriptional regulation. The translation process can be divided into initiation, elongation, termination, and ribosome recycling, but translation initiation is the most important stage for the regulation of protein synthesis [82, 83]. This involves the assembly of elongation-competent 80S ribosomes (each consisting of a large 60S subunit and a small 40S subunit) and requires at least nine eukaryotic initiation factors (eIFs), numbered eIF1-9 [83]. The 40S subunit first binds eIF3 and an eIF2-GTP-initiator transfer RNA ternary complex, then the 48S initiation complex is assembled with the help of many eIFs via numerous steps [83]. eIF5B (*LOC_Os05g51500*), which was most strongly expressed in both rice lines at low temperature, is essential for the binding of 60S subunits and dissociation of eIF1, eIF1A, eIF3 and residual eIF2–GDP [84]. Ribosomes play a basic housekeeping role in global translation. Four genes encoding ribosomal proteins (*LOC_Os08g03450*, *LOC_Os01g64090*, *LOC_Os01g69950* and *LOC_Os07g12250*, encoding L37, L1p/L10e, L27 and L24, respectively) were up-regulated in both rice lines at low temperature. Previous studies have shown that the expression of the 60S ribosomal protein L37 and two 40S ribosomal proteins of Arabidopsis increases during low-temperature treatment, indicating that they might enhance translation or support proper ribosome assembly and functions at low temperatures [85]. Thus, eIF5B and the four ribosomal proteins that we found to be induced by low temperature may enhance translation of *Ugp1* transcripts in low-temperature conditions. In addition, there are recent indications that ribosomal protein genes have regulatory roles in plant development [86, 87].

Aminoacyl-tRNA synthetases (ARSs) are housekeeping enzymes that are essential for protein synthesis and have many other functions including participation in DNA replication, RNA splicing and eukaryotic cell processes related to cytokine function and cell cycle control [88]. Several ARS genes are reportedly involved in gametogenesis and embryo development in Arabidopsis [89]. Seven ARSs were up-regulated in Co27LT relative to Co27HT, and may be essential for the accumulation of UGPase protein in Co27LT. Another gene induced in Co27LT, which may play a role in translational termination, encodes the eukaryotic elongation factor Tu (eEF-Tu, *LOC_Os04g20220*). Homologous proteins are the most abundant proteins in most bacterial cells and have established roles in translation and other processes, including cell shape maintenance [90]. Nascent polypeptide chains should be released from peptidyl-tRNA when the ribosome encounters a stop signal in the mRNA being translated. Peptide chain release factor proteins play an essential role in this process [91]. *LOC_Os05g19630* encodes a peptide chain release factor protein and was up-regulated in Co27LT. Thus, together with the *eEF-Tu* gene it may play an important role in the termination of UGPase protein translation.

In summary, eIF5B, four ribosomal proteins (L37, L1p/L10e, L27 and L24), seven ARSs, eEF-Tu and a peptide chain release factor protein may play significant translational roles in TGMS (Table 2).

### qRT-PCR analysis

**Figure 6:**
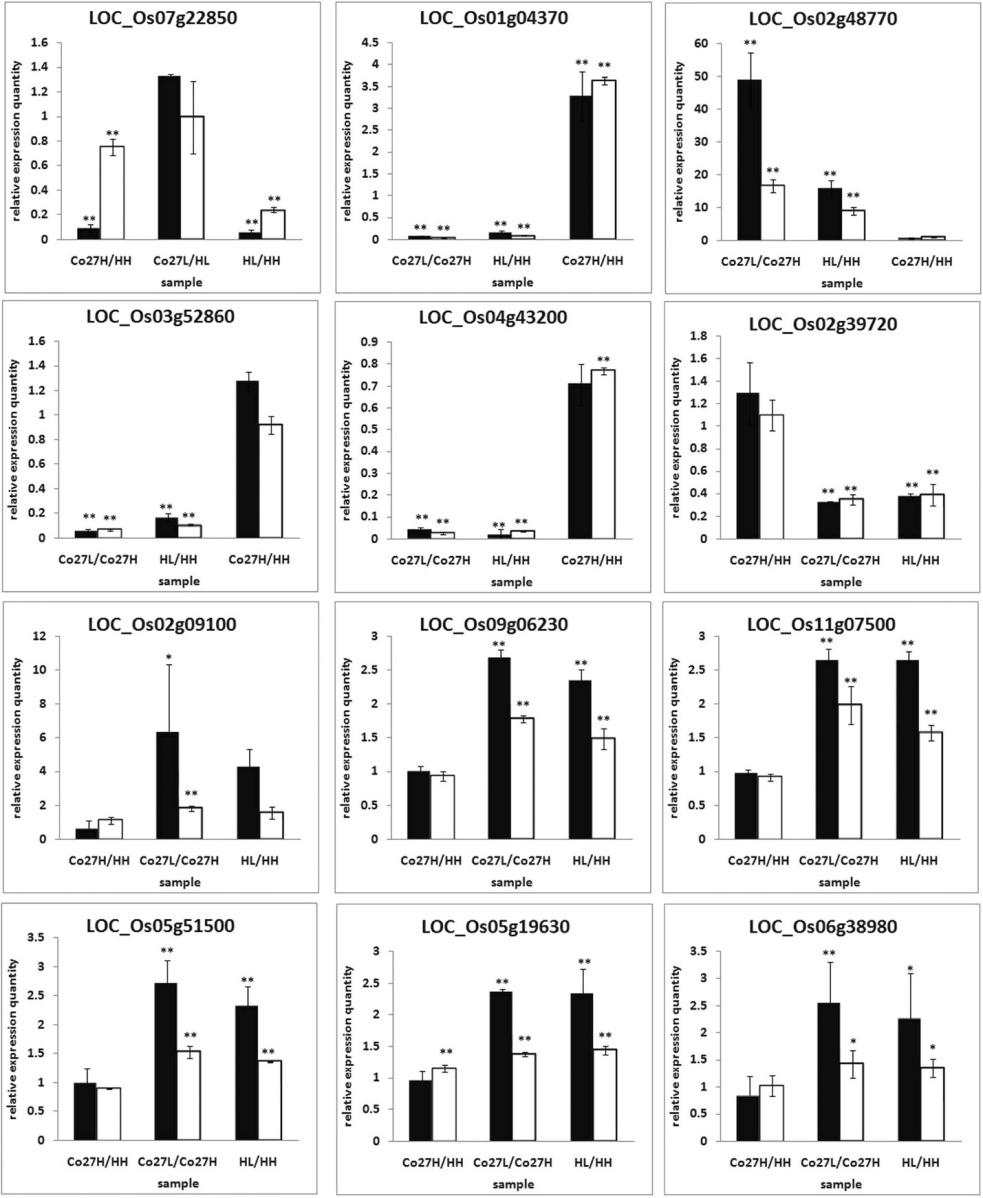
**Quantitative RT-PCR verification of expression patterns of differentially expressed genes detected in the microarray analysis.** The black bars represent the strength of expression of the genes in indicated samples according to the microarray data while the white bars represent corresponding quantitative RT-PCR results. Significant differences are indicated by *P < 0.05, **P < 0.01 (Student’s t-test). The sample names are the same as those in Table 2.

To validate our microarray data, qRT-PCR analysis was performed on 12 randomly selected DEGs that are probably important for pollen development, low temperature responses or TGMS (Figure 6). The results, in terms of relative expression levels among the four sample types, were consistent with the microarray data, especially for LOC_Os01g04370, LOC_Os03g52860, LOC_Os04g43200, LOC_Os02g39720, LOC_Os09g06230 and LOC_Os11g07500, although differences in expression levels of other genes were smaller than those detected in the microarray analysis. Overall, we conclude that our microarray data are highly credible.

## Conclusions

Our microarray analysis revealed that temperature strongly influences global gene expression, indicating that plants may fine-tune their expression profiles in adaptive responses to temperature changes that optimize their growth and reproduction processes. Numerous genes involved in pollen development, particularly genes required for cell wall biosynthesis, were repressed in TGMS-Co27 plants at high temperature. We also identified a cluster of temperature-inducible genes (designated cluster 15 here) that appear to be vital for sterility transition. Many enriched genes of this cluster are associated with the meiosis stage, indicating that low temperature may be a common regulator of fertility alterations in PGMS/TGMS rice lines. In combination with previous findings, our results also indicate that RS-containing zinc finger proteins, PTBs, DEAD/DEAH box RNA helicases, ZOS, polyadenylate-binding proteins, and some other RRM-containing proteins play key post-transcriptional roles in the male sterility of TGMS-Co27 plants, while eIF5B, several ribosomal proteins (L37, L1p/L10e, L27 and L24), ARSs, eEF-Tu and a peptide chain release factor protein play important translational roles. However, further studies are needed to verify the functions of these candidate genes and improve our understanding of TGMS mechanisms.

### Availability of supporting data

The complete microarray data sets supporting the results presented herein have been deposited in the NCBI’s Gene Expression Omnibus [92] and are accessible using GEO Series accession number GSE56779.

## Electronic supplementary material

Additional file 1: **Primers used in all PCR experiments.** (XLS 30 KB)

Additional file 2: Figure S1: RNA gel blot analysis of *Ugp1* transcript levels in meiosis-stage florets. The RNA gel blot was hybridized with corresponding *Ugp1* probes. Arrowheads indicate: (1) the unprocessed longer-than-full-length transcript; (2) endogenous *Ugp1* mRNA; (3) silencing-related RNA degradation intermediates. Loading of equal amounts of RNA was confirmed by ethidium bromide staining. H1493 refers to a mixture (in equal proportions) of total RNAs from H1493 plants grown at high temperature and low temperature. Co27HT and Co27LT refer to TGMS-Co27 plants grown at high temperature and low temperature, respectively. **Figure S2.** GO slimes of functional categorization of DEGs related to stress responses. The abscissa represents the number of corresponding genes in clusters 10 and 15 (genes down-regulated and up-regulated by different environmental conditions). **Figure S3.** Heatmap of the expression of TGMS-related genes in the four sample types. H1493HT and H1493LT refer to H1493 plants grown at high temperature and low temperature, respectively. Co27HT and Co27LT refer to TGMS-Co27 plants grown at high temperature and low temperature, respectively. **Figure S4.** Semi-quantitative RT-PCR analysis of the expression and splicing patterns of selected serine/arginine-rich (*SR*) genes. The names of the genes are shown on the left of each panel. An equal quantity of template in each reaction was verified by amplifying a constitutively expressed actin. **Table S1.** Pollen development-related genes that were repressed in TGMS-Co27 plants at high temperature. HH and HL refer to H1493 plants grown at high temperature and low temperature, respectively, while Co27H and Co27L refer to TGMS-Co27 plants grown at high temperature and low temperature, respectively. Significant changes (p-value < 0.05; fold change ≥ 2) are indicated by boldface. (PDF 506 KB)

Additional file 3: **All differentially expressed genes, shown in Figure** 2 **C, detected by microarray analyses.** (XLS 7 MB)

Additional file 4: **Gene ontology (GO) categories and enrichment of the up-regulated and down-regulated DEGs between TGMS-Co27 and H1493 plants grown at high temperature (Co27HT vs. H1493HT).** (XLS 2 MB)

Additional file 5: **Pollen development-related genes.** This file lists pollen development-related DEGs and their gene ontology (GO) categories. (XLS 38 KB)

Additional file 6: **Results of series-cluster analysis of DEGs between TGMS-Co27 plants grown at high temperature and at low temperature, showing the genes in all 25 clusters.** The profile number indicates the cluster number. (XLS 5 MB)

Additional file 7: **Gene ontology (GO) categories and enrichment of the DEGs included in the six main clusters shown in Figure** 4 **.** (XLS 9 MB)

Additional file 8: **Details of the 156 DEGs, including GO categories and cluster numbers, apparently involved in the TGMS of TGMS-Co27.** (XLS 269 KB)

Additional file 9: **Details of the co-expression network of the 156 TGMS-related genes, showing attributes of all 156 genes, including their degrees and “k core-scores”.** Fifty-eight with k core-scores exceeding 20 are considered to be key regulators of male sterility in TGMS-Co27. Profiles of these 58 genes were analyzed and the key regulators were classed into two groups by their working level. (XLS 340 KB)

## Abbreviations

TGMS: Thermosensitive genic male sterile
PGMS: Photoperiod sensitive genic male sterile
CMS: Cytoplasmic male sterile
DEGs: Differentially expressed genes
qRT-PCR: Quantitative RT-PCR
H1493HT: H1493 plants grown at high temperature
Co27HT: TGMS-Co27 plants grown at high temperature
H1493LT: H1493 plants grown at low temperature
Co27LT: TGMS-Co27 plants grown at low temperature
PCA: Principal component analysis
HCL: Hierarchical clustering
FC: Fold change
GO: Gene ontology
JA: Jasmonic acid
MMC: Microspore mother cell.

## References

[1] Chase CD, Ribarits A, Heberle-Bors E, Pua EC, Davey MR (2010). Male sterility. Plant Developmental Biology - Biotechnological Perspectives.

[2] Perez-Prat E, van Lookeren Campagne MM (2002). Hybrid seed production and the challenge of propagating male-sterile plants. Trends Plant Sci.

[3] Irish VF (2010). The flowering of Arabidopsis flower development. Plant J.

[4] Honys D, Twell D (2003). Comparative analysis of the Arabidopsis pollen transcriptome. Plant Physiol.

[5] Hennig L, Gruissem W, Grossniklaus U, Köhler C (2004). Transcriptional programs of early reproductive stages in Arabidopsis. Plant Physiol.

[6] Pina C, Pinto F, Feijó JA, Becker JD (2005). Gene family analysis of the Arabidopsis pollen transcriptome reveals biological implications for cell growth, division control, and gene expression regulation. Plant Physiol.

[7] Wijeratne AJ, Zhang W, Sun Y, Liu W, Albert R, Zheng Z, Oppenheimer DG, Zhao D, Ma H (2007). Differential gene expression in Arabidopsis wild-type and mutant anthers: insights into anther cell differentiation and regulatory networks. Plant J.

[8] Zheng B, Wu X, Ge X, Deng X, Grosser JW, Guo W (2012). Comparative transcript profiling of a male sterile cybrid pummelo and its fertile type revealed altered gene expression related to flower development. PLoS One.

[9] Scott RJ, Spielman M, Dickinson HG (2004). Stamen structure and function. Plant Cell.

[10] Xu X, Jeffrey SR (1998). Efficiency and technical progress in traditional and modern agriculture: evidence from rice production in China. Agric Econ.

[11] Virmani S (1994). Prospects of hybrid rice in the tropics and subtropics. Hybrid rice technology: new developments and future prospects Manila (Philippines): International Rice Research Institute p.

[12] Li J, Yuan L (2010). Hybrid rice: genetics, breeding, and seed production. Plant Breeding Reviews.

[13] Yuan L (1990). Progress of two-line system hybrid rice breeding. Sci Agric Sin.

[14] Chen R, Pan Y, Wang Y, Zhu L, He G (2009). Temperature-sensitive splicing is an important molecular regulation mechanism of thermosensitive genic male sterility in rice. Chin Sci Bull.

[15] Li J, Xin Y, Yuan L (2009). Hybrid rice technology development: ensuring China's food security. International Food Policy Research Institute (IFPRI).

[16] Wang B, Xu W, Wang J, Wu W, Zheng H, Yang Z, Ray JD, Nguyen HT (1995). Tagging and mapping the thermo-sensitive genic male-sterile gene in rice (*Oryza sativa* L.) with molecular markers. Theor Appl Genet.

[17] Yamaguchill Y, Ikedazl R, Hirasawazl H, Minamin M, Ujiharall A (1997). Linkage analysis of thermosensitive genic male sterility gene, tms-2 in rice (*Oryza sativa* L.). Breeding Sci.

[18] Subudhi PK, Borkakati RP, Virmani SS, Huang N (1997). Molecular mapping of a thermosensitive genetic male sterility gene in rice using bulked segregant analysis. Genome.

[19] Dong NV, Subudhi PK, Luong PN, Quang VD, Quy TD, Zheng H, Wang B, Nguyen HT (2000). Molecular mapping of a rice gene conditioning thermosensitive genic male sterility using AFLP, RFLP and SSR techniques. Theor Appl Genet.

[20] Wang Y, Xing Q, Deng Q, Liang F, Yuan L, Weng M, Wang B (2003). Fine mapping of the rice thermo-sensitive genic male-sterile gene tms5. Theor Appl Genet.

[21] Lee D, Chen L, Suh H (2005). Genetic characterization and fine mapping of a novel thermo-sensitive genic male-sterile gene tms6 in rice (*Oryza sativa* L.). Theor Appl Genet.

[22] Jia J, Zhang D, Li C, Qu X, Wang S, Chamarerk V, Nguyen HT, Wang B (2001). Molecular mapping of the reverse thermo-sensitive genic male-sterile gene (rtms1) in rice. Theor Appl Genet.

[23] Sheng Z, Wei X, Shao G, Chen M, Song J, Tang S, Luo J, Hu Y, Hu P, Chen L (2013). Genetic analysis and fine mapping of tms9, a novel thermosensitive genic male-sterile gene in rice (*Oryza sativa* L.). Plant Breed.

[24] Liu N, Shan Y, Wang F, Xu C, Peng K, Li X, Zhang Q (2001). Identification of an 85-kb DNA fragment containing pms1, a locus for photoperiod-sensitive genic male sterility in rice. Mol Genet Genomics.

[25] Zhang Q, Shen B, Dai X, Mei M, Saghai Maroof MA, Li Z (1994). Using bulked extremes and recessive class to map genes for photoperiod-sensitive genic male sterility in rice. Proc Natl Acad Sci U S A.

[26] Lu Q, Li X, Guo D, Xu C, Zhang Q (2005). Localization of pms3, a gene for photoperiod-sensitive genic male sterility, to a 28.4-kb DNA fragment. Mol Genet Genomics.

[27] Xu J, Wang B, Wu Y, Du P, Wang J, Wang M, Yi C, Gu M, Liang G (2011). Fine mapping and candidate gene analysis of ptgms2-1, the photoperiod-thermo-sensitive genic male sterile gene in rice (*Oryza sativa* L.). Theor Appl Genet.

[28] Zhou Y, Zhang X, Xue Q (2011). Fine mapping and candidate gene prediction of the photoperiod and thermo-sensitive genic male sterile gene pms1(t) in rice. J Zhejiang Univ Sci B.

[29] Peng H, Zhang Z, Wu B, Chen X, Zhang G, Zhang Z, Wan B, Lu Y (2008). Molecular mapping of two reverse photoperiod-sensitive genic male sterility genes (rpms1 and rpms2) in rice (*Oryza sativa* L.). Theor Appl Genet.

[30] Ding J, Lu Q, Ouyang Y, Mao H, Zhang P, Yao J, Xu C, Li X, Xiao J, Zhang Q (2012). A long noncoding RNA regulates photoperiod-sensitive male sterility, an essential component of hybrid rice. Proc Natl Acad Sci U S A.

[31] Zhou H, Liu Q, Li J, Jiang D, Zhou L, Wu P, Lu S, Li F, Zhu L, Liu Z, Chen L, Liu Y, Zhuang C (2012). Photoperiod- and thermo-sensitive genic male sterility in rice are caused by a point mutation in a novel noncoding RNA that produces a small RNA. Cell Res.

[32] Zhang H, Xu C, He Y, Zong J, Yang X, Si H, Sun Z, Hu J, Liang W, Zhang D (2013). Mutation in CSA creates a new photoperiod-sensitive genic male sterile line applicable for hybrid rice seed production. Proc Natl Acad Sci U S A.

[33] Zhou H, Zhou M, Yang Y, Li J, Zhu L, Jiang D, Dong J, Liu Q, Gu L, Zhou L, Feng M, Qin P, Hu X, Song C, Shi J, Song X, Ni E, Wu X, Deng Q, Liu Z, Chen M, Liu Y, Cao X, Zhuang C (2014). RNase Z^S1^ processes *Ub*_*L40*_ mRNAs and controls thermosensitive genic male sterility in rice. Nat Commun.

[34] Chen R, Zhao X, Shao Z, Wei Z, Wang Y, Zhu L, Zhao J, Sun M, He R, He G (2007). Rice UDP-glucose pyrophosphorylase1 is essential for pollen callose deposition and its cosuppression results in a new type of thermosensitive genic male sterility. Plant Cell.

[35] Feng J, Lu Y, Liu X, Xu X (2001). Pollen development and its stages in rice (*Oryza sativa* L.). Zhongguo shuidao kexue.

[36] Itoh J-I, Nonomura K-I, Ikeda K, Yamaki S, Inukai Y, Yamagishi H, Kitano H, Nagato Y (2005). Rice plant development: from zygote to spikelet. Plant Cell Physiol.

[37] Smyth GK (2004). Linear models and empirical bayes methods for assessing differential expression in microarray experiments. Stat Appl Genet Mol Biol.

[38] Alexa A, Rahnenführer J, Lengauer T (2006). Improved scoring of functional groups from gene expression data by decorrelating GO graph structure. Bioinformatics.

[39] Pfaffl MW (2001). A new mathematical model for relative quantification in real-time RT–PCR. Nucleic Acids Res.

[40] Prieto C, Risueño A, Fontanillo C, De Las Rivas J (2008). Human gene coexpression landscape: confident network derived from tissue transcriptomic profiles. PLoS One.

[41] Barabasi A-L, Oltvai ZN (2004). Network biology: understanding the cell's functional organization. Nat Rev Genet.

[42] Ravasz E, Somera AL, Mongru DA, Oltvai ZN, Barabási A-L (2002). Hierarchical organization of modularity in metabolic networks. Science.

[43] Chen F, Zhu H, Zhou L, Li J, Zhao L, Wu S, Wang J, Liu W, Chen Z (2010). Genes related to the very early stage of ConA-induced fulminant hepatitis: a gene-chip-based study in a mouse model. BMC Genomics.

[44] Yun K-Y, Park MR, Mohanty B, Herath V, Xu F, Mauleon R, Wijaya E, Bajic VB, Bruskiewich R, de los Reyes BG (2010). Transcriptional regulatory network triggered by oxidative signals configures the early response mechanisms of japonica rice to chilling stress. BMC Plant Biol.

[45] Zhang T, Zhao X, Wang W, Pan Y, Huang L, Liu X, Zong Y, Zhu L, Yang D, Fu B (2012). Comparative transcriptome profiling of chilling stress responsiveness in two contrasting rice genotypes. PLoS One.

[46] Liechti R, Farmer EE (2002). The jasmonate pathway. Science.

[47] Feys BJ, Benedetti CE, Penfold CN, Turner JG (1994). Arabidopsis mutants selected for resistance to the phytotoxin coronatine are male sterile, insensitive to methyl jasmonate, and resistant to a bacterial pathogen. Plant Cell.

[48] Ishiguro S, Kawai-Oda A, Ueda J, Nishida I, Okada K (2001). The DEFECTIVE IN ANTHER DEHISCENCE1 gene encodes a novel phospholipase A1 catalyzing the initial step of jasmonic acid biosynthesis, which synchronizes pollen maturation, anther dehiscence, and flower opening in Arabidopsis. Plant Cell.

[49] Stone BA, Clarke AE (1992). Chemistry and Biology of 1,3-β-Glucans.

[50] McCormick S (1993). Male gametophyte development. Plant Cell.

[51] Wang W, Liu Z, Guo Z, Song G, Cheng Q, Jiang D, Zhu Y, Yang D (2011). Comparative transcriptomes profiling of photoperiod-sensitive male sterile rice Nongken 58S during the male sterility transition between short-day and long-day. BMC Genomics.

[52] Renaut J, Hausman J-F, Bassett C, Artlip T, Cauchie H-M, Witters E, Wisniewski M (2008). Quantitative proteomic analysis of short photoperiod and low-temperature responses in bark tissues of peach (Prunus persica L. Batsch). Tree Genetics & Genomes.

[53] Jaglo-Ottosen KR, Gilmour SJ, Zarka DG, Schabenberger O, Thomashow MF (1998). Arabidopsis CBF1 overexpression Induces COR genes and enhances freezing tolerance. Science.

[54] Wang Q, Guan Y, Wu Y, Chen H, Chen F, Chu C (2008). Overexpression of a rice *OsDREB1F* gene increases salt, drought, and low temperature tolerance in both *Arabidopsis* and rice. Plant Mol Biol.

[55] Kaul ML (1988). Male Sterility in Higher Plants.

[56] Xiao G, Yuan L (1997). Effects of water temperature on male sterility of the thermo-sensitive genic male sterile (TGMS) rice lines under the simulated low air temperature conditions appeared occasionally in high summer. Chinese J Rice Sci.

[57] Zeng H, Zhang Z, Yuan S, Li Y, Zhang D (1993). Studies on the thermo-sensitive period of fertility alteration in photoperiod-sensitive genic male sterile rice. J Huazhong U.

[58] Brunner M, Schafmeier T (2006). Transcriptional and post-transcriptional regulation of the circadian clock of *cyanobacteria* and *Neurospora*. Genes Dev.

[59] Diernfellner AC, Schafmeier T, Merrow MW, Brunner M (2005). Molecular mechanism of temperature sensing by the circadian clock of *Neurospora crassa*. Genes Dev.

[60] Huang Y, McNeil GP, Jackson FR (2014). Translational regulation of the DOUBLETIME/CKIδ/ϵ kinase by LARK contributes to circadian period modulation. PLoS Genet.

[61] Stuart JM, Segal E, Koller D, Kim SK (2003). A gene-coexpression network for global discovery of conserved genetic modules. Science.

[62] Floris M, Mahgoub H, Lanet E, Robaglia C, Menand B (2009). Post-transcriptional regulation of gene expression in plants during abiotic stress. Int J Mol Sci.

[63] Lareau LF, Green RE, Bhatnagar RS, Brenner SE (2004). The evolving roles of alternative splicing. Curr Opin Struct Biol.

[64] Stamm S, Ben-Ari S, Rafalska I, Tang Y, Zhang Z, Toiber D, Thanaraj TA, Soreq H (2005). Function of alternative splicing. Gene.

[65] Wahl MC, Will CL, Lührmann R (2009). The spliceosome: design principles of a dynamic RNP machine. Cell.

[66] Valadkhan S, Jaladat Y (2010). The spliceosomal proteome: at the heart of the largest cellular ribonucleoprotein machine. Proteomics.

[67] Lorković ZJ, Wieczorek Kirk DA, Lambermon MHL, Filipowicz W (2000). Pre-mRNA splicing in higher plants. Trends Plant Sci.

[68] Zhang P, Deng H, Xiao F, Liu Y (2013). Alterations of alternative splicing patterns of Ser/Arg-rich (SR) genes in response to hormones and stresses treatments in different ecotypes of rice (*Oryza sativa*). J Integr Agric.

[69] Isshiki M, Tsumoto A, Shimamoto K (2006). The serine/arginine-rich protein family in rice plays important roles in constitutive and alternative splicing of pre-mRNA. Plant Cell.

[70] Valcárcel J, Green MR (1996). The SR protein family: pleiotropic functions in pre-mRNA splicing. Trends Biochem Sci.

[71] Wang S, Okamoto T (2009). Involvement of polypyrimidine tract-binding protein (PTB)-related proteins in pollen germination in Arabidopsis. Plant Cell Physiol.

[72] Stauffer E, Westermann A, Wagner G, Wachter A (2010). Polypyrimidine tract-binding protein homologues from Arabidopsis underlie regulatory circuits based on alternative splicing and downstream control. Plant J.

[73] Palusa SG, Ali GS, Reddy ASN (2007). Alternative splicing of pre-mRNAs of Arabidopsis serine/arginine-rich proteins: regulation by hormones and stresses. Plant J.

[74] Western TL, Cheng Y, Liu J, Chen X (2002). HUA ENHANCER2, a putative DExH-box RNA helicase, maintains homeotic B and C gene expression in Arabidopsis. Development.

[75] Lange H, Sement FM, Gagliardi D (2011). MTR4, a putative RNA helicase and exosome co-factor, is required for proper rRNA biogenesis and development in Arabidopsis thaliana. Plant J.

[76] Fuller-Pace FV (2006). DExD/H box RNA helicases: multifunctional proteins with important roles in transcriptional regulation. Nucleic Acids Res.

[77] Agarwal P, Arora R, Ray S, Singh A, Singh V, Takatsuji H, Kapoor S, Tyagi A (2007). Genome-wide identification of C2H2 zinc-finger gene family in rice and their phylogeny and expression analysis. Plant Mol Biol.

[78] Sharma R, Agarwal P, Ray S, Deveshwar P, Sharma P, Sharma N, Nijhawan A, Jain M, Singh A, Singh V, Khurana J, Tyagi A, Kapoor S (2012). Expression dynamics of metabolic and regulatory components across stages of panicle and seed development in indica rice. Funct Integr Genomics.

[79] Xiao H, Tang J, Li Y, Wang W, Li X, Jin L, Xie R, Luo H, Zhao X, Meng Z, He G, Zhu L (2009). *STAMENLESS 1*, encoding a single C2H2 zinc finger protein, regulates floral organ identity in rice. Plant J.

[80] Chekanova JA, Shaw RJ, Belostotsky DA (2001). Analysis of an essential requirement for the poly(A) binding protein function using cross-species complementation. Curr Biol.

[81] Mangus DA, Evans MC, Jacobson A (2003). Poly (A)-binding proteins: multifunctional scaffolds for the post-transcriptional control of gene expression. Genome Biol.

[82] Sonenberg N, Hinnebusch AG (2009). Regulation of translation initiation in eukaryotes: mechanisms and biological targets. Cell.

[83] Jackson RJ, Hellen CUT, Pestova TV (2010). The mechanism of eukaryotic translation initiation and principles of its regulation. Nat Rev Mol Cell Biol.

[84] Pestova TV, Lomakin IB, Lee JH, Choi SK, Dever TE, Hellen CUT (2000). The joining of ribosomal subunits in eukaryotes requires eIF5B. Nature.

[85] Kim KY, Park SW, Chung YS, Chung CH, Kim JI, Lee JH (2004). Molecular cloning of low-temperature-inducible ribosomal proteins from soybean. J Exp Bot.

[86] Byrne ME (2009). A role for the ribosome in development. Trends Plant Sci.

[87] Horiguchi G, Van Lijsebettens M, Candela H, Micol JL, Tsukaya H (2012). Ribosomes and translation in plant developmental control. Plant Sci.

[88] Francklyn C, Perona JJ, Puetz J, Hou Y-M (2002). Aminoacyl-tRNA synthetases: versatile players in the changing theater of translation. RNA.

[89] Berg M, Rogers R, Muralla R, Meinke D (2005). Requirement of aminoacyl-tRNA synthetases for gametogenesis and embryo development in Arabidopsis. Plant J.

[90] Defeu Soufo HJ, Reimold C, Linne U, Knust T, Gescher J, Graumann PL (2010). Bacterial translation elongation factor EF-Tu interacts and colocalizes with actin-like MreB protein. Proc Natl Acad Sci U S A.

[91] Buckingham RH, Grentzmann G, Kisselev L (1997). Polypeptide chain release factors. Mol Microbiol.

[92] Edgar R, Domrachev M, Lash AE (2002). Gene expression omnibus: NCBI gene expression and hybridization array data repository. Nucleic Acids Res.

