# Supplementary material for: Genes associated with thermosensitive genic male sterility in rice identified by comparative expression profiling
Source: BMC Genomics. 2014 Dec 16;15(1):1114. doi: 10.1186/1471-2164-15-1114 (PMC4320516; doi:10.1186/1471-2164-15-1114)
Supplement: Supplementary file 2 — Additional file 2: Figure S1: RNA gel blot analysis of Ugp1 transcript levels in meiosis-stage florets. The RNA gel blot was hybridized with corresponding Ugp1 probes. Arrowheads indicate: (1) the unprocessed longer-than-full-length transcript; (2) endogenous Ugp1 mRNA; (3) silencing-related RNA degradation intermediates. Loading of equal amounts of RNA was confirmed by ethidium bromide staining. H1493 refers to a mixture (in equal proportions) of total RNAs from H1493 plants grown at high temperature and low temperature. Co27HT and Co27LT refer to TGMS-Co27 plants grown at high temperature and low temperature, respectively. Figure S2. GO slimes of functional categorization of DEGs related to stress responses. The abscissa represents the number of corresponding genes in clusters 10 and 15 (genes down-regulated and up-regulated by different environmental conditions). Figure S3. Heatmap of the expression of TGMS-related genes in the four sample types. H1493HT and H1493LT refer to H1493 plants grown at high temperature and low temperature, respectively. Co27HT and Co27LT refer to TGMS-Co27 plants grown at high temperature and low temperature, respectively. Figure S4. Semi-quantitative RT-PCR analysis of the expression and splicing patterns of selected serine/arginine-rich (SR) genes. The names of the genes are shown on the left of each panel. An equal quantity of template in each reaction was verified by amplifying a constitutively expressed actin. Table S1. Pollen development-related genes that were repressed in TGMS-Co27 plants at high temperature. HH and HL refer to H1493 plants grown at high temperature and low temperature, respectively, while Co27H and Co27L refer to TGMS-Co27 plants grown at high temperature and low temperature, respectively. Significant changes (p-value < 0.05; fold change ≥ 2) are indicated by boldface. (PDF 506 KB) [file 12864_2014_6904_MOESM2_ESM.pdf]

Figure S1

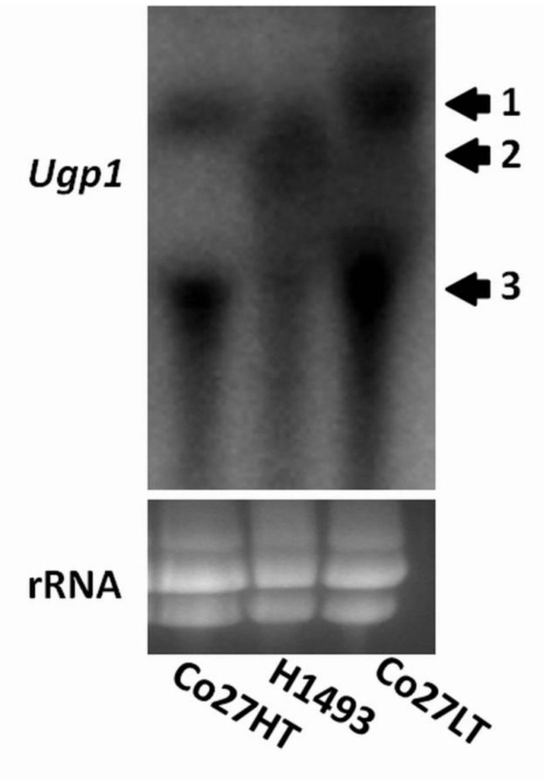

Figure S2

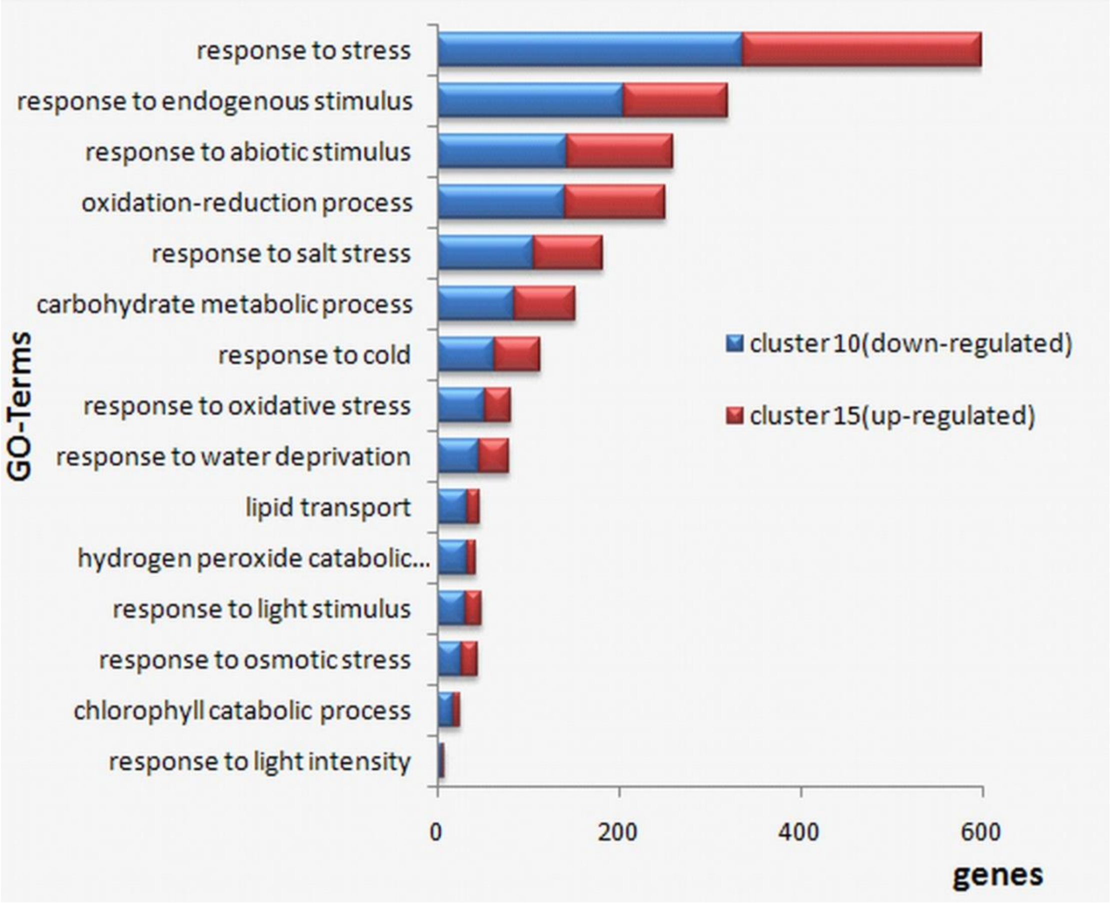
GO-Terms
response to stress
response to endogenous stimulus
response to abiotic stimulus
oxidation-reduction process
response to salt stress
carbohydrate metabolic process
response to cold
response to oxidative stress
response to water deprivation
lipid transport
hydrogen peroxide catabolic...
response to light stimulus
response to osmotic stress
chlorophyll catabolic process
response to light intensity
cluster 10(down-regulated)
cluster 15(up-regulated)
0
200
400
600
genes

Figure S3

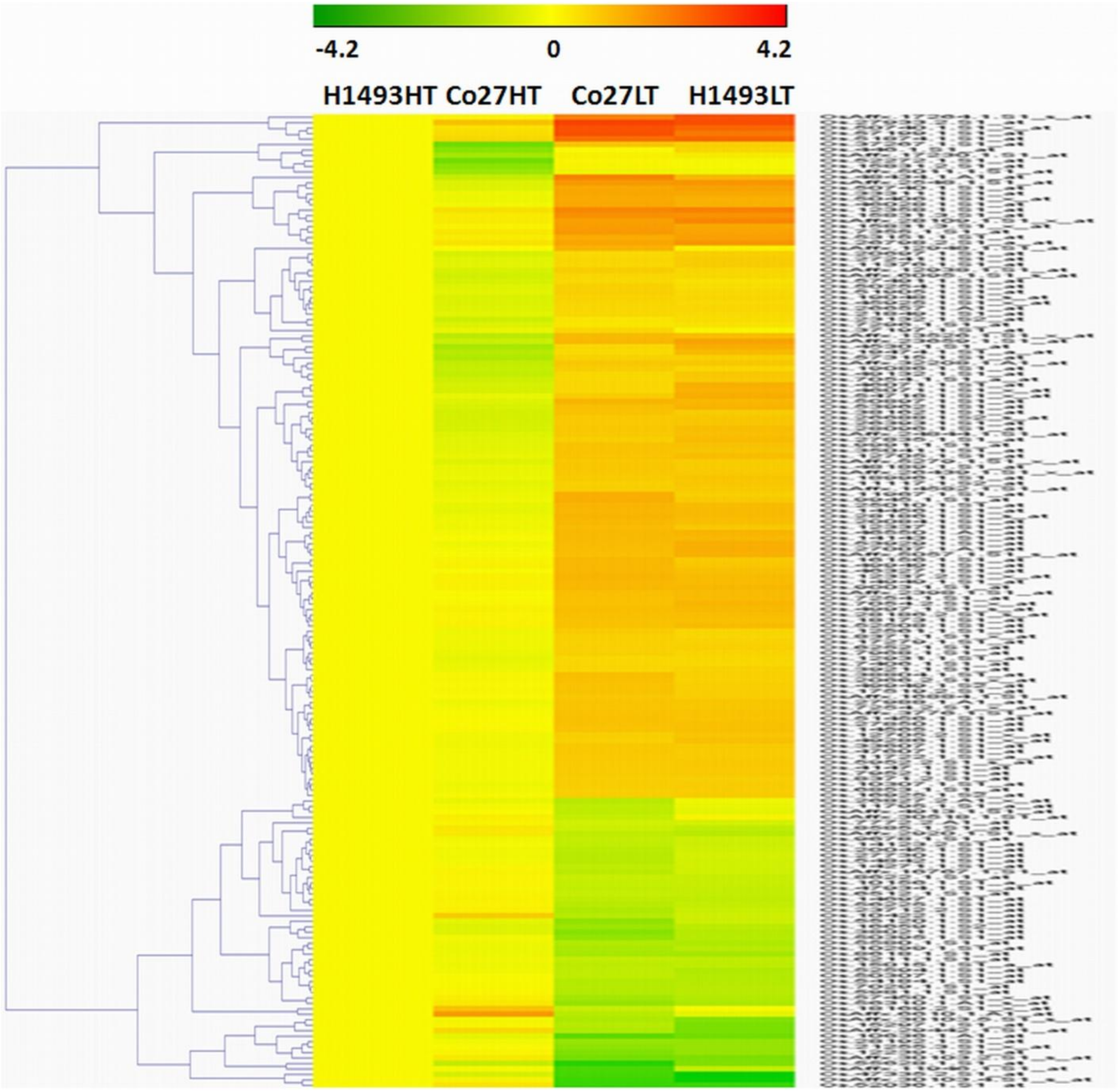

Figure S4

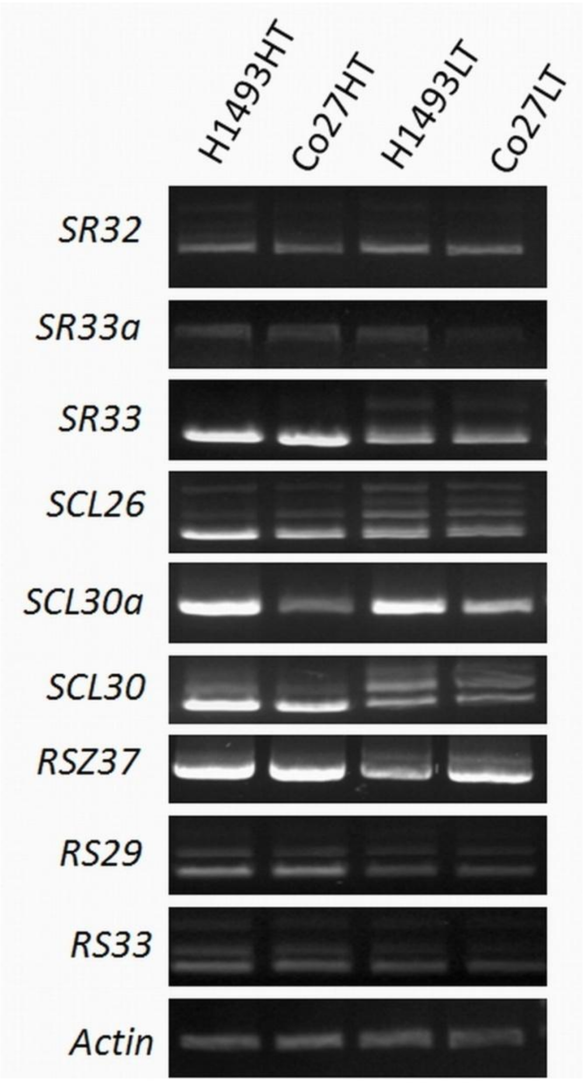
H1493HT
Co27HT
H1493LT
Co27LT
SR32
SR33a
SR33
SCL26
SCL30a
SCL30
RSZ37
RS29
RS33
Actin

SUPPLEMENTAL FIGURE LEGENDS

Figure S1. RNA gel blot analysis of *Ugp1* transcript levels in meiosis-stage florets.

The RNA gel blot was hybridized with corresponding *Ugp1* probes. Arrowheads indicate: (1) the unprocessed longer-than-full-length transcript; (2) endogenous *Ugp1* mRNA; (3) silencing-related RNA degradation intermediates. Loading of equal amounts of RNA was confirmed by ethidium bromide staining. H1493 refers to a mixture (in equal proportions) of total RNAs from H1493 plants grown at high temperature and low temperature. Co27HT and Co27LT refer to TGMS-Co27 plants grown at high temperature and low temperature, respectively.

Figure S2. GO slimes of functional categorization of DEGs related to stress responses.

The abscissa represents the number of corresponding genes in clusters 10 and 15 (genes down-regulated and up-regulated by different environmental conditions).

Figure S3. Heatmap of the expression of TGMS-related genes in the four sample types.

H1493HT and H1493LT refer to H1493 plants grown at high temperature and low temperature, respectively. Co27HT and Co27LT refer to TGMS-Co27 plants grown at high temperature and low temperature, respectively.

Figure S4. Semi-quantitative RT-PCR analysis of the expression and splicing patterns of selected serine/arginine-rich (*SR*) genes.

The names of the genes are shown on the left of each panel. An equal quantity of template in each reaction was verified by amplifying a constitutively expressed actin.

Table S1. Pollen development-related genes that were repressed in TGMS-Co27 plants at high temperature.

QuerySymbol	probeID	Description	E-Value	SubjectSymbol	logFC_ Co27H/ HH	logFC_ Co27L/ HL	logFC_ HL/HH
**LOC_Os01g03670**	Os.20305.1.S1_at	dihydroflavonol-4-reductase, putative, expressed	1.00E-116	AT1G68540.2	**-1.87**	-1.63	-1.39
**LOC_Os01g48440**	Os.19374.1.S1_at	glycosyltransferase family 43 protein, putative, expressed	3.00E-62	AT1G27600.2	**-1.53**	-0.24	**1.46**
**LOC_Os02g02560**	Os.2370.1.S1_at	UTP--glucose-1-phosphate uridylyltransferase, putative, expressed	0	AT5G17310.2	**-1.39**	-0.42	-0.44
**LOC_Os06g08380**	OsAffx.27509.1.S2_at	1,3-beta-glucan synthase component domain containing protein, expressed	0	AT2G13680.1	**-2.59**	-1.44	-0.54
**LOC_Os07g22850**	OsAffx.28544.2.S1_x_at	chalcone and stilbene synthases, putative, expressed	1.00E-156	AT4G34850.1	**-3.4**	0.41	**-4.05**
**LOC_Os09g38030**	Os.10118.1.S1_at	UTP--glucose-1-phosphate uridylyltransferase, putative, expressed	0	AT5G17310.2	**-2.01**	-0.73	0.21

HH and HL refer to H1493 plants grown at high temperature and low temperature, respectively, while Co27H and Co27L refer to TGMS-Co27 plants grown at high temperature and low temperature, respectively. Significant changes ($p < 0.05$; fold change ≥ 2) are indicated by boldface.
